# Isolation and genomic characterization of chicken infectious anemia virus in Jiangsu province of China during 2020–2022

**DOI:** 10.3389/fvets.2024.1378120

**Published:** 2024-03-14

**Authors:** Jun Zhang, Li Ma, Tuofan Li, Quan Xie, Zhimin Wan, Aijian Qin, Jianqiang Ye, Hongxia Shao, Shengnan Wang

**Affiliations:** ^1^Key Laboratory of Jiangsu Preventive Veterinary Medicine, Key Laboratory for Avian Preventive Medicine, Ministry of Education, College of Veterinary Medicine, Yangzhou University, Yangzhou, China; ^2^Jiangsu Co-Innovation Center for Prevention and Control of Important Animal Infectious Diseases and Zoonoses, Yangzhou, China; ^3^Joint International Research Laboratory of Agriculture and Agri-Product Safety, The Ministry of Education of China, Yangzhou University, Yangzhou, China; ^4^Institutes of Agricultural Science and Technology Development, Yangzhou University, Yangzhou, China

**Keywords:** chicken infectious anemia virus (CIAV), isolation, complete genome, VP1, molecular characteristics

## Abstract

As an immunosuppressive disease virus, chicken infectious anemia virus (CIAV) mainly infects chickens, causing aplastic anemia and systemic lymphoid tissue atrophy. In recent years, the prevalence of CIAV in the poultry industry globally has caused huge economic losses. In this study, a total of 223 clinical samples, including anal swabs, tissues, blood, and vaccines, were collected from 19 broiler farms or breeding companies in Jiangsu province, with symptoms of significant anemia and immunosuppression during 2020–2022. Among them, 75 samples (75/223, 33.6%) were positive for CIAV in polymerase chain reaction (PCR) test, and 20 CIAV strains were successfully isolated. The phylogenetic trees of the 20 isolates and 42 CIAV strains deposited in GenBank formed four distinct groups (A-D). And the isolates mainly belonged to Group A but with high genetic diversity. Analysis for VP1 indicated that these isolates possess key characteristics of highly pathogenic strains. Meanwhile, VP2 and VP3 were much conserved with much fewer mutations compare to VP1. The above epidemiological study of CIAV provides novel insights into molecular characterization of CIAV and lays the foundation for developing efficient strategies for control of CIAV in China.

## Introduction

As an important immunosuppressive virus, chicken infectious anemia virus (CIAV) mainly causes aplastic anemia and systemic lymphoid tissue atrophy in chicks, while increasing susceptibility to other pathogens and decreasing immune response to vaccines (1–4). CIAV is classified into genus *Gyrovirus*, the family *Anelloviridae* by the International Committee on Taxonomy of Viruses (ICTV) (5). The single strand DNA of CIAV genome contains three overlapped open reading frames (ORFs) that encoding three viral proteins, namely VP1 (51.2 kDa), VP2 (24 kDa), and VP3 (13.6 kDa) (6). Among them, VP1 is the only capsid protein and the main immunogenic protein with neutralizing epitopes of CIAV (7). VP2 plays a role in viral assembly and has dual-specificity phosphatase activity, similar to Torque teno virus (TTV)-encoded ORF2 (8, 9). VP3 protein, also known as apoptin (10), can selectively mediate cell death (11, 12), and it is essential for viral replication (13, 14).

Since CIAV was first isolated and identified in Japan in 1979 (15), it has spread worldwide. Subsequently, CIAV was first reported and isolated from Heilongjiang province in China in 1992 (16). With the development of modern poultry industry globally, CIAV commercial attenuated vaccines are widely used in breeding flocks, but not in China. In China, CIAV infection and co-infection with other viruses, such as Marek’s disease virus (MDV), reticuloendotheliosis virus (REV), infectious bursal disease virus (IBDV), subgroup J avian leukosis virus (ALV-J), fowl adenovirus (FAdV), avian reovirus (ARV), H9N2 influenza virus (H9N2), and infectious bronchitis virus (IBV), are common in commercial chickens, which can aggravate the diseases (4, 17–21). Recent serological surveys of CIAV in chicken farms revealed high seropositivity rate in Zhejiang, Jiangsu, and Anhui provinces of China (22). Of note, CIAV can reduce the immune effect of vaccines and enhance the virulence of live attenuated vaccines, resulting in immune failure (4). CIAV could also enhance the pathogenicity of other pathogens and further induce high morbidity and mortality (4, 17–21). Thus, continuously following up on the incidence of CIAV infection in chicken flocks is extremely important for the prevention of CIAV.

In this study, we investigated the CIAV epidemiology of clinical samples from 19 broiler farms or breeding companies in Jiangsu province of China during 2020–2022. The PCR for CIAV showed that 75 samples (75/223, 33.6%) were positive and 20 CIAV strains were successfully isolated. Phylogenetic analysis for complete genome and amino acid sequences of VP1 revealed that all these 20 isolates belonged to highly virulent strains and could be clustered into four major groups (A-D). Our study provides novel insights into the epidemiology and efficient strategies for prevention and control of CIAV in China.

## Materials and methods

### Clinical samples

A total of 223 clinical samples (including anal swabs, tissues, blood, and vaccines) were received from 19 broiler farms or breeding companies in Jiangsu province (From Yancheng, Nantong, Taizhou, Changzhou, and Lianyungang Cities) during 2020–2022. These chickens showed anemia, thymus atrophy, and poor production performance, and PCR was mainly used to detect CIAV. The anal swabs and tissues samples were homogenized with phosphate-buffered saline (PBS), and supernatants were collected by centrifugation at 12000 rpm/min for 30 min at 4°C. Then, the collected supernatants were treated with 5 × penicillin and streptomycin for 45 min at 37°C and filtered through 0.22 μm filters for virus isolation. Blood and vaccine samples were directly inoculated into cells as shown below.

### Polymerase chain reaction for detection of CIAV

The genomic DNA was extracted from the clinical samples using a TIANamp Genomic DNA Kit (TIANGEN, Beijing, China) and stored at-20°C. For the detection of CIAV, the PCR was performed with specific primers of VP3 gene listed in Table 1. The PCR reaction volume was 20 μL containing 10 μL of 2 × Taq Plus Master Mix II (Dye Plus) (Vazyme, Nanjing, China), 1 μL of each primer (10 μM), 7 μL of double-distilled water (ddH_2_O), and 1 μL of the DNA template. The PCR cycling conditions for the VP3 gene amplifications were as follows: 1 cycle of 95°C for 5 min, 35 cycles of 95°C for 30 s, 56°C for 30 s, and 72°C for 30 s, followed by a final extension step of 72°C for 10 min.

**Table 1:** Primers for PCR detection of CIAV VP3, linearization of pcDNA3.1, and complete genome amplification of CIAV.

Name	Sequence (5′-3′)	Length (bp)
CIAV-VP3-F	ATGAACGCTCTCCAAGAAGATAC	366
CIAV-VP3-R	TTACAGTCTTATACGCCTTTTTGCG	
pcDNA3.1-F	GAATTCTGCAGATATCCAGCACAGTG	5,403
pcDNA3.1-R	GCTCGGTACCAAGCTTAAGTTTAAACG	
pc-CIAV-F	AGCTTGGTACCGAGCGCATTCCAAGTGGTTACTATTC	2,328
pc-CIAV-R	ATATCTGCAGAATTCGATTGTGCGATAAAGCCATTTGCT	

### Cell culture, virus isolation, and identification

The MDCC-MSB-1 cells (kept in our laboratory) were cultured at 37°C with 5% CO_2_ in the cell incubator. The supernatants of the PCR-positive samples were inoculated into MDCC-MSB-1 cells for virus isolation. The supernatants were collected at 3–4 days post-infection (dpi) and detected by PCR with the primers CIAV-VP3-F/R. To amplify the complete genome of the isolates, a pair of primers was designed for PCR amplification as listed in Table 1.

### Sequence and phylogenetic analysis

The pcDNA3.1 vector was linearized by PCR with primers listed in Table 1, and then the complete genome of the isolates with homologous arms was amplified with Phanta Super-Fidelity DNA Polymerase (Vazyme, China) and then cloned into pcDNA3.1 using a ClonExpress II One Step Cloning Kit (Vazyme, China). The PCR positive clones were sequenced by Genecfps (Wuxi, China) via sanger sequencing. The phylogenetic trees were constructed using neighbor-joining method using MEGA6.1 software with 1,000 bootstrap replicates based on the nucleotide sequences of the complete genome and amino acid sequences of VP1 of the isolates and the reference strains deposited in GenBank (Table 2). The deduced amino acid sequences of VP1, VP2, VP3, and untranslated regions (UTRs) of the isolates compared with the reference strains were also conducted using the Clustal W method in the MegAlign program by the Lasergene 7.0 software for molecular features.

**Table 2:** The sequence information of 20 CIAV isolates and CIAV reference strains used in this study.

Strains	Year	Origin	GenBank accession No.
JSCZ20T1P6	2020	Jiangsu, China	PP354989
JS2007Chen	2020	Jiangsu, China	PP354990
JS2007Yao	2020	Jiangsu, China	PP354991
JS2009Shi	2020	Jiangsu, China	PP354992
JS210317	2021	Jiangsu, China	PP354993
JS210422	2021	Jiangsu, China	PP354994
JS2105A	2021	Jiangsu, China	PP354995
JS2105B	2021	Jiangsu, China	PP354996
JSCZ2105	2021	Jiangsu, China	PP354997
JSTZ2105	2021	Jiangsu, China	PP354998
JS210724	2021	Jiangsu, China	PP354999
JS2107Liu	2021	Jiangsu, China	PP355000
JS211939	2021	Jiangsu, China	PP355001
JS211940	2021	Jiangsu, China	PP355002
JS211949	2021	Jiangsu, China	PP355003
JSNT21Sun	2021	Jiangsu, China	PP355004
JSNT21Zeng	2021	Jiangsu, China	PP355005
JS221021	2022	Jiangsu, China	PP355006
JS22101782	2022	Jiangsu, China	PP355007
JS22101784	2022	Jiangsu, China	PP355008
3–1	2003	Malaysia	AF390038.1
5-IM201910	2019	China	OQ116656.1
10-AH201911	2019	Anhui, China	OQ116658.1
20-SD201911	2019	Shandong, China	OQ116673.1
26P4	2007	The Netherland	D10068.1
98D02152	2016	USA	AF311892.2
704	1996	Australia	U65414.1
1860TW	2018	Taiwan, China	MT799769.1
AB1K	2016	Turkey	MT259319.1
AH4	2005	Anhui, China	DQ124936.1
BD-3	2004	Bangladesh	AF395114.1
C368	2001	Japan	AB046589.1
C369	2001	Japan	AB046590.1
CIA-1	1996	USA	L14767.1
CIAV89-69	2011	South Korea	JF507715.1
CQ21313	2021	China	OP038327.1
Cux-1	2008	Germany	M55918.1
Cuxhaven	1993	Germany	M81223.1
Del-Ros	2000	USA	AF313470.1
FJ21425	2021	Fujian, China	OP038331.1
FJ21821	2021	Fujian, China	OP038334.1
GD-1-12	2012	Guangdong, China	JX260426.1
GD-103	2016	Guangdong, China	KU050678.1
GX1804	2018	Guangxi, China	MK484615.1
GX2020-D3	2020	Guangxi, China	MW579761.1
GX21124	2021	Guangxi, China	OP038345.1
GXC060821	2006	Guangxi, China	JX964755.1
HLJ14101	2014	Heilongjiang, China	KY486136.1
HLJ15170	2015	Heilongjiang, China	KY486144.1
JL14023	2014	Jilin, China	KY486145.1
JS2020-PFV	2020	Jangsu, China	MW234428.1
JS15165	2015	Jangsu, China	KY486152.1
LF4	2005	Hebei, China	AY839944.1
LN15169	2015	China	KY486154.1
LY-1	2016	Shandong, China	KX447636.1
Pigeon-CIAV-1906	2019	China	MT536347.1
SDSPF2020	2020	Shandong, China	MW660821.1
SH16	2005	Shanghai, China	DQ141671.1
SMSC-1	2003	Malaysia	AF285882.1
SMSC-1P60	2003	Malaysia	AF390102.1
TJBD33	2005	Tianjin, China	AY843527.2
TR20	1999	Japan	AB027470.1

## Results

### Prevalence of CIAV in Jiangsu Province of China during 2020–2022

**Figure 1:**
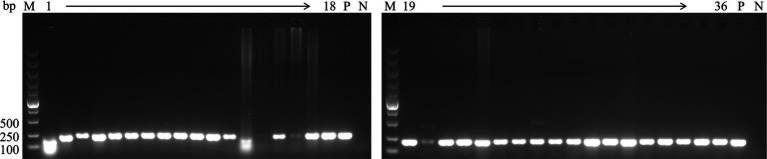
PCR detection for CIAV of clinical samples. The genomic DNA extracted from the clinical samples were detected by PCR using specific primers targeting *VP3* gene of CIAV listed in Table 1. Lane M: Super DNA Marker; Lane P: positive control; Lane N: negative control; Lane 1–36: clinical samples with strong CIAV-positive in PCR detection.

**Figure 2:**
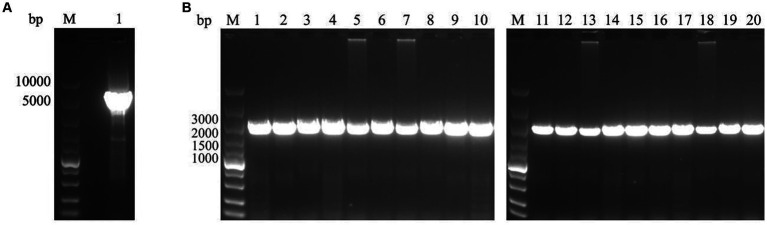
PCR amplification for linearization of pcDNA3.1 vector and the complete genome of 20 CIAV isolates. **(A)** The pcDNA3.1 vector was linearized by PCR with specific primers pcDNA3.1-F/*R. lane* M: Super DNA Marker; Lane 1: the linearized pcDNA3.1. **(B)** The complete genome of 20 isolates with homologous arm of pcDNA3.1 was amplified by PCR with specific primers pc-CIAV-F/*R. lane* M: Super DNA Marker; Lane 1–20: Total of 20 CIAV isolates from clinical samples.

To investigate the epidemiology of CIAV in Jiangsu province of China, 223 clinical samples from 19 broiler flocks or breeding companies in Jiangsu province of China during 2020–2022 were tested. PCR result showed that 75 samples (75/223, 33.6%) were CIAV-positive. As shown in Figures 1, 36 clinical samples were CIAV-positive. These data demonstrated that CIAV was prevalent in Jiangsu province of China during 2020–2022. In addition, we also tested the co-infection status of these 36 samples, and the results showed that there are dual, triple, and quintuple co-infections of CIAV with ALV, FAdV, MDV, REV, and ARV (Supplementary Table S1). To isolate the clinical samples, 36 samples with strong CIAV-positive bands were inoculated into MDCC-MSB-1 cells for virus isolation, and 20 strains were successfully isolated for complete genome sequencing. The amplified complete genome of the 20 isolates with homologous arm of pcDNA3.1 were ligated with linearized pcDNA3.1 vector and further sequenced (Figure 2).

### Phylogenetic analysis of complete genome and VP1 of CIAV

Among the 20 CIAV isolates, the complete genome of the 17 isolates were 2,298 nucleotides in length, whereas three isolates had deletion or insertion in UTRs. The isolate JSNT21Sun (2,297 bp) had one nucleotide deletion, the isolate JS211949 (2,296 bp) had a deletion of two nucleotides and the isolate JSCZ2105 (2,299 bp) had one nucleotide insertion. The 20 isolates showed high nucleotide sequence identity with the three main vaccine strains, Cux-1 (96.5–98.0%, Germany), 26P4 (96.3–99.5%, The Netherland), and Del-Ros (97.9–98.6%, United States). The nucleotide similarity between the 20 isolates was high (96.3–100%), with the lowest 96.3% (JS2105B and JS2107Liu) and the highest 100% (JS2105A and JLTZ2105). Notably, the JS2105A was isolated from an attenuated live vaccine, and the JLTZ2105 sharing 100% nucleotide identity with the JS2105A was isolated from chicken liver tissue, suggesting that JLTZ2105 was derived from the vaccine contaminated with JS2105A. In addition, the deduced amino acid sequences of VP1 of the 20 isolates also shared high similarity with the three main vaccine strains Cux-1 (97.1–98.7%), 26P4 (97.6–99.6%), and Del-Ros (97.8–99.3%), and had high similarity (97.3–100%) with each other.

**Figure 3:**
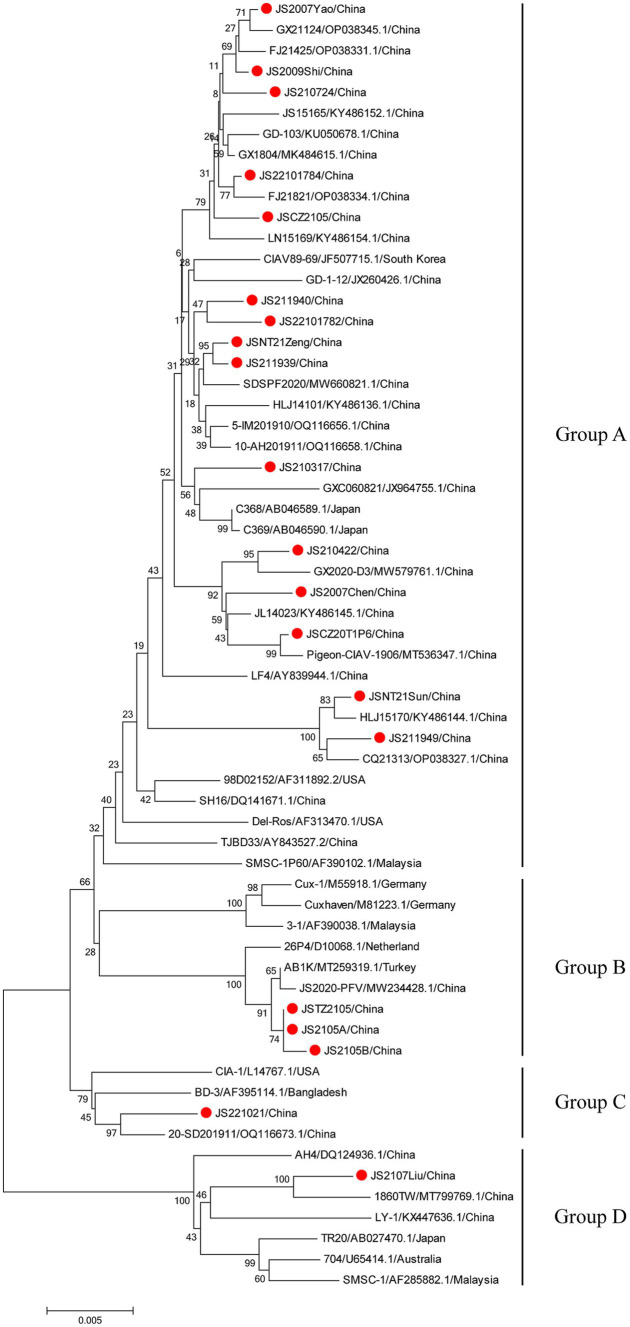
Phylogenetic tree analysis based on the nucleotide sequences of complete genome. The phylogenetic tree was constructed using the neighbor-joining method (1,000 bootstraps) with MEGA6. The 20 isolates in this study were indicated by the red closed circle.

**Figure 4:**
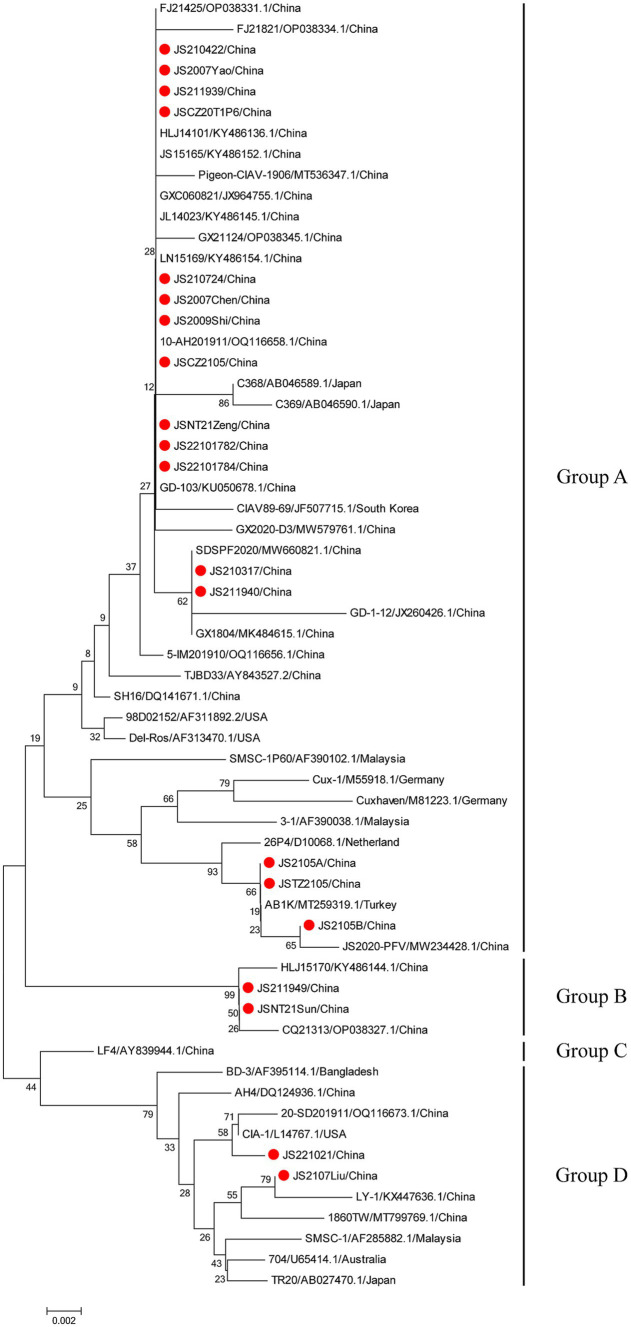
Phylogenetic tree analysis based on the amino acid sequences of VP1. The phylogenetic tree was constructed using the neighbor-joining method (1,000 bootstraps) with MEGA6. The 20 isolates in this study were indicated by the red closed circle.

The phylogenetic trees were conducted based on the nucleotide sequences of the complete genome and the deduced amino acid sequences of VP1 of the 20 isolates and the 42 reference strains such as LF4 (China,), Cux-1, CIA (United States), and TR20 (Japan). As shown in Figure 3, the 62 strains were clustered into four major groups (A-D) based on the genome sequence. Fifteen of the 20 isolates belonged to Asian branch Group A and three (JS2105A, JS2105B, and JSTZ2105) of the 20 isolates belonged to Group B, the isolates JS221021 and JS2107Liu were clustered into Group C and Group D, respectively. As shown in Figure 4, similar to complete genome, the amino acid sequences of VP1of 62 strains were also clustered into four major groups (A-D). Among them, 16 of the 20 isolates belonged to Asian branch Group A, JS211949 and JSNT21Sun were clustered into Group B, and other two isolates (JS2107Liu and JS221021) belonged to Group D. The above data showed that the isolates in this study mainly belonged to Group A.

### Amino acid substitution in VP1, VP2, and VP3 of CIAV

**Figure 5:**
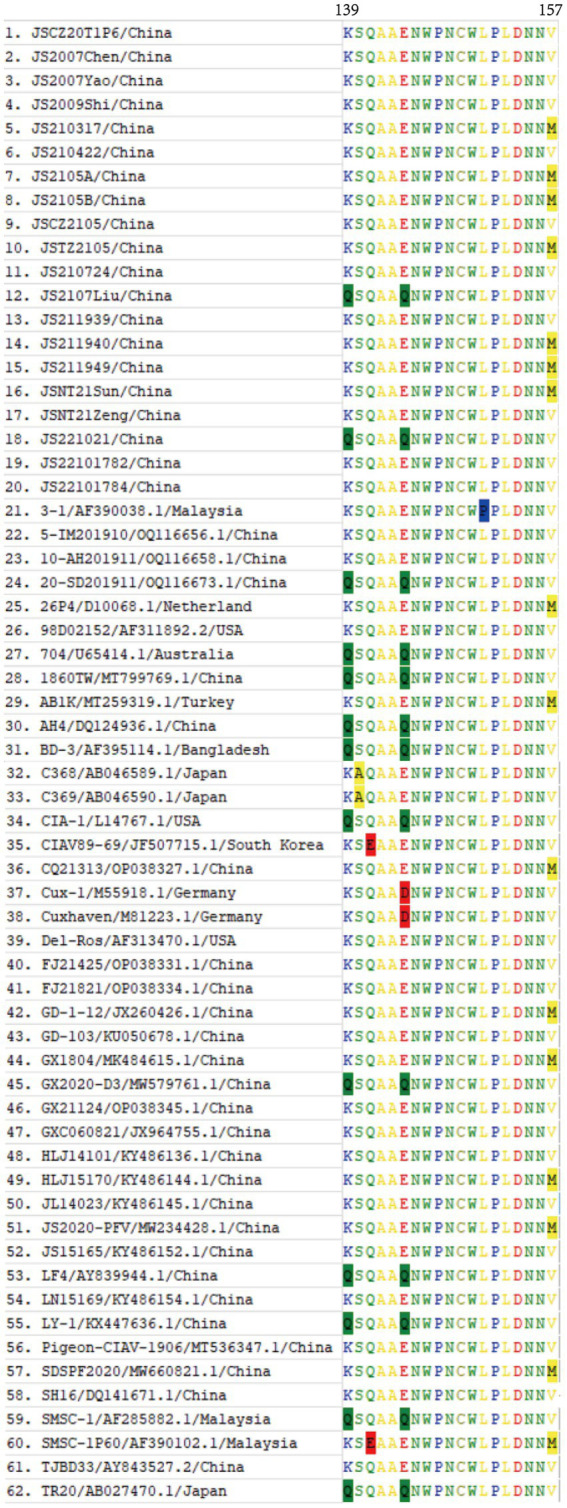
Amino acid alignment of the VP1 hypervariable region (aa 139–157) of 20 isolates and 42 CIAV reference strains available in GenBank. The substitutions of amino acids of VP1 occurring at sites 139, 140, 141, 144, 151, and 157 of 62 strains were highlighted with background color.

In comparison with the 42 reference strains, the deduced amino acid sequences of VP1 of the 20 isolates in this study had 18 substitutions (V25I, V75I, T89K, G92D, M97L, L125I, K139Q, E144Q, V157M, E254G, S287T, S287A, A290P, Q294H, G370S, G370A, G370T, I376L, S413A, V436I, and S447T), and the mutation probability was 4%. As described in Figure 5, in the hypervariable region (aa 139–157) of VP1, JS2107Liu and JS221021 had two amino acid substitutions (K139Q and E144Q), which had an influence on the rate of replication or dissemination of infection in MDCC-MSB-1 cells (23). Seven isolates (JS210317, JS2105A, JS2105B, JSTZ2105, JS211940, JS211949, and JSNT21Sun) had one amino acid substitution (V157M). The known virulence-determining sites in VP1 of CIAV were shown in Table 3. The 20 isolates presented Q at amino acid sites 141 and 394, indicating all the isolates possessed sequence characteristics of highly virulent strains (24). In addition, at amino acid site 75, 18 isolates presented V instead of I as the majority virulent strains, except JS2107Liu and JS221021. At amino acid site 89, only JS2105B was K instead of T in the low virulent strains. Five isolates (JS2105A, JS2105B, JSTZ2105, JS2107Liu, and JS221021) presented I instead of L as the majority virulent strains shown at amino acid site 125. At amino acid sites 139 and 144, only JS2107Liu and JS221021 were Q, which is consist with the majority virulent strains, whereas the other 18 isolates were K or E. Seven isolates (JS210317, JS2105A, JS2105B, JSTZ2105, JS211940, JS211949, and JSNT21Sun) presented M instead of V as the low virulent strains at amino acid site 157. At amino acid site 287, six isolates (JS2105A, JS2105B, JSTZ2105, JS2107Liu, JS211949, and JSNT21Sun) presented T as the majority virulent strains, JS221021 had an A with rare reports, and the other 13 isolates presented S as the low virulent strains. Notably, amino acid site 370 in VP1 had three substitutions (G370S, G370A, and G370T). The substitutions of key amino acid sites in VP1 between the live attenuated vaccine sample JS2105A and reference strain AB1K were completely consistent, revealing a potential genetic evolutionary relationship.

**Table 3:** The known virulence determining sites in VP1 of the isolates.

Isolates/Amino acid sites	75	89	125	139	141	144	157	287	394
Majority virulent strains	V	T	I	Q	Q	Q	V	T	Q
Low virulent strains	I	A	L	K	E	E	M	S	H
JSCZ20T1P6	V	T	L	K	Q	E	V	S	Q
JS2007Chen	V	T	L	K	Q	E	V	S	Q
JS2007Yao	V	T	L	K	Q	E	V	S	Q
JS2009Shi	V	T	L	K	Q	E	V	S	Q
JS210317	V	T	L	K	Q	E	M	S	Q
JS210422	V	T	L	K	Q	E	V	S	Q
JS2105A	V	T	I	K	Q	E	M	T	Q
JS2105B	V	K	I	K	Q	E	M	T	Q
JSCZ2105	V	T	L	K	Q	E	V	S	Q
JSTZ2105	V	T	I	K	Q	E	M	T	Q
JS210724	V	T	L	K	Q	E	V	S	Q
JS2107Liu	I	T	I	Q	Q	Q	V	T	Q
JS211939	V	T	L	K	Q	E	V	S	Q
JS211940	V	T	L	K	Q	E	M	S	Q
JS211949	V	T	L	K	Q	E	M	T	Q
JSNT21Sun	V	T	L	K	Q	E	M	T	Q
JSNT21Zeng	V	T	L	K	Q	E	V	S	Q
JS221021	I	T	I	Q	Q	Q	V	A	Q
JS22101782	V	T	L	K	Q	E	V	S	Q
JS22101784	V	T	L	K	Q	E	V	S	Q

Each amino acid is labeled with one color for a better comparison.

In addition, only two substitutions were found in VP2 (D169G: JS2105A, JS2105B, and JSTZ2105; G177A: JS2107Liu), with a mutation probability of 0.9%. Eight substitutions were detected in VP3 (L4P, P9Q, R23Q, P24L, L25S, V73A, S103N, and P112H), with a mutation probability of 6.6% (Table 4).

**Table 4:** The amino acid mutation sites in VP3 of the isolates.

Isolates/Amino acid sites	4	9	23	24	25	73	103	112
JSCZ20T1P6	L	P	R	P	L	V	S	P
JS2007Chen	L	P	R	P	L	V	S	P
JS2007Yao	L	P	R	P	L	V	S	P
JS2009Shi	L	P	R	P	L	V	S	P
JS210317	L	Q	R	P	L	V	S	P
JS210422	L	P	R	P	L	V	S	P
JS2105A	P	P	Q	P	L	A	N	P
JS2105B	P	P	Q	P	L	A	N	H
JSCZ2105	L	P	R	P	S	V	S	P
JSTZ2105	P	P	Q	P	L	A	N	P
JS210724	L	P	R	P	L	V	S	P
JS2107Liu	L	P	R	P	L	V	S	P
JS211939	L	P	R	L	L	V	S	P
JS211940	L	P	R	P	L	V	S	P
JS211949	L	P	R	P	L	V	S	P
JSNT21Sun	L	P	R	P	L	V	S	P
JSNT21Zeng	L	P	R	P	L	V	S	P
JS221021	L	P	R	P	L	V	S	P
JS22101782	L	P	R	P	L	V	S	P
JS22101784	L	P	R	P	L	V	S	P

Each amino acid is labeled with one color for a better comparison.

### Molecular characterization of UTRs from the 20 CIAV isolates

**Figure 6:**
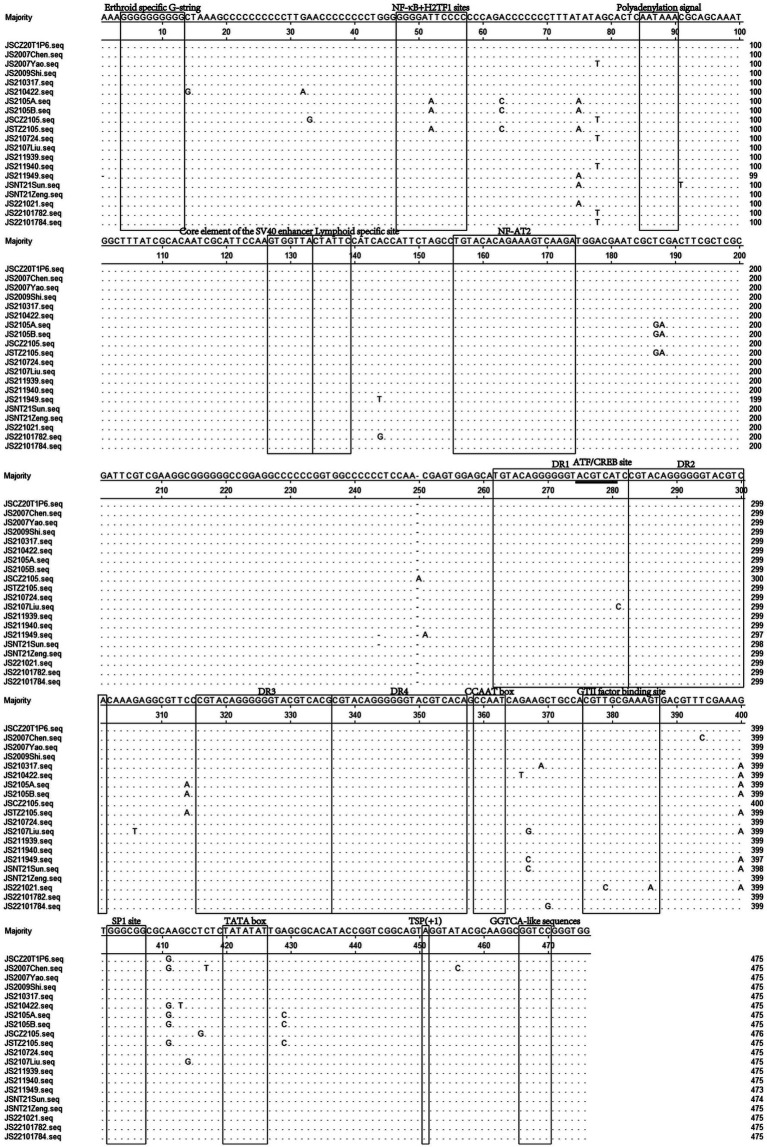
Sequence alignment of the UTRs nucleotide sequences of 20 CIAV isolates. The sequences in black frames are the motifs of transcriptional regulatory elements in this study. Conserved nucleotide sequences were indicated with dots and differences were indicated with letters.

The UTR of CIAV is consists of approximately 300 centrally distributed nucleotides, containing more than a dozen conserved sequences known to be associated with replication and transcriptional regulation (25–28). In this study, the Clustal W method was used to analyze the nucleotide sequences of UTRs of 20 CIAV isolates, and the alignment result showed that the UTRs of the isolates contained DNA conserved regions with high G + C content (nucleotide homology 97.5–100%). Further analysis showed that the deletion and insertion in the UTRs of the isolates were not on the transcription factor binding motifs, and most of the motifs located in the UTRs were the same, such as Erthroid specific G-string, Polyadenylation signal, Core element of the SV40 enhancer, Lymphoid specific site, NF-AT2, SP1 site, and TATA box (Figure 6). However, there were also several single base mutations located in some motifs, such as NFκB+H2TF1 sites and GTII factor binding sites (Figure 6). Whether the mutations of these two motifs affect the binding of corresponding transcription factors and further have impacts on the replication of CIAV still need to be studied. In addition, transcription factor binding site analysis conducted by NSITE demonstrated that a tandem array consisting of four DR regions was found 1 nt upstream of the “CCAAT” box in the UTRs of all isolates (Figure 6). The ATF/CREB binding sites (ACGTCA) in the four DRs are similar to imperfect hormone response element half-sites (AGGTCA), and a cluster of GGTCA-like sequences was found downstream of the transcription initiation point (TSP) (Figure 6).

## Discussion

Since Yuasa et al. first isolated CIAV in Japan in 1979 (15), CIAV has spread globally and caused huge economic losses to the poultry industry worldwide (25, 29). CIAV was first reported and isolated from Heilongjiang province in China in 1992 by Cui et al. (16). At present, from the perspective of the antigenicity of isolated strains worldwide, it is generally considered that there is only one serotype of CIAV (30, 31). However, the sequences of CIAV isolates in different regions were different, and the virulence changed greatly, which has become one of the important pathogens threatening the poultry industry worldwide (32).

In this study, we investigated CIAV infection in clinical samples from 19 broiler farms or breeding companies in Jiangsu province from 2020 to 2022 and compared the sequences of the obtained isolates with related reference strains. The investigation found that the positive rate of CIAV in 223 samples was 33.6% and 20 CIAV strains from different farms or company were successfully isolated. The nucleotide similarity of between the 20 isolates was 96.3–100%, whereas the identity of the complete genomic nucleotide sequence of CIAV isolated from the same farm or company were higher than 99%, except for JSNT21Sun and JSNT21Zeng (98.3%). Of note, although JSNT21Sun and JSNT21Zeng were clustered into Group A, their genetic evolutionary distance was higher when compared to other strains from the same field. Thus, it is necessary to be alert to the emergence of new recombinant viruses when strains with genetic distance exist in the same breeding farm. In addition, the co-infections of CIAV with other viruses, such as ALV-A/J/K, FAdV-4, MDV, and ARV, were also detected (Supplementary Table S1). These indicated that the co-infection of immunosuppressive pathogens still common in China, which should be taken more seriously. Currently, CIAV infection is generally prevented and controlled by vaccination in chickens, but no commercial vaccine is available in China (33). Thus, CIAV was very prevalent in China during the past years (34–37). Moreover, a study reported that a highly pathogenic CIAV strain was isolated from 2-month-old chickens for the first time in China, indicating that the epidemic CIAV strains have the potential to break age resistance with increasing pathogenicity (38).

As the only structural protein of CIAV, VP1 gene encodes the viral capsid and is the main immunogenic protein with neutralizing epitopes (7). VP1 is also the most variable protein in different CIAV strains (25, 39, 40). Moreover, the known variations in the hypervariable region of VP1 (139–151 aa) can impact on the protein structure prediction of VP1 (23, 41, 42). In this study, mutations were found in VP1 (Figure 4). Further research is needed to determine whether these mutations in VP1 could affect its antigenicity. VP2 is a non-structural protein in CIAV and acts as a scaffold protein to participate in the correct folding of VP1 during virion assembly (8, 43), assisting the production of neutralizing antibodies (44). Of note, VP2 is the most conserved protein in CIAV, with only two substitutions (D169G and G177A) in the isolates of this study. In addition, VP2 can also induce apoptosis (10), and has dual-specificity phosphatase activity, similar to TTV-encoded ORF2 (8, 9). Whether the two substitutions in VP2 impact its function still needs to be further studied. As another non-structural protein, VP3 is known as apoptin (10), which can selectively mediate cell death (11, 12), and it is essential for viral replication (13, 14). However, only eight substitutions (L4P, P9Q, R23Q, P24L, L25S, V73A, S103N, and P112H) were found in VP3 of the 20 isolates, which is much less than that of VP1. And the impact of these substitutions on the function of VP3 needs to be explored in the future. In addition, whether the mutations of NF-κB + H2TF1 sites and GTII factor binding sites in the UTRs of the isolates have impacts on the replication of CIAV should also be further studied.

A previous study found that amino acid site 394 of VP1 is a genetic determinant of pathogenicity in CIAV, and the mutation of Q to H can decrease the virulence of CIAV (24). Herein, all 20 isolates presented Q at amino acid site 394, indicating that all the isolates were highly likely to be highly pathogenic strains as well. Renshaw et al. (23) found that 139–151 aa is the hypervariable region in VP1, and the construction of various chimeric viruses of Cux-1 and CIA-1 proved that Q139 and/or Q144 impact the replication rate of CIAV and its infective ability in MDCC-MSB-1 cells. Meanwhile, the cell tropism of CIAV is related to amino acid sites 139, 144, and some upstream amino acid sites in VP1 (23). In this study, isolates JS2107Liu and JS221021 had Q at amino acid sites 139 and 144, representing the majority virulent strains, indicating that their replication in MDCC-MSB-1 cells or transmission in chickens may be different from the other 18 isolates. Notably, it is rarely reported that CIAV strains present A at amino acid site 287 (37), however, isolate JS221021 here had an A as well, which revealed that the mutation of rare amino acid site has a tendency to increase. All these demonstrate that the isolated strains with high sequence diversity got a high possibility of high pathogenicity.

It is noteworthy that the isolate JLTZ2105 sharing 100% nucleotide homology with JS2105A in chickens was derived from the attenuated vaccine contaminated with CIAV, which is consistent with the widespread presence of low pathogenic CIAV in SPF chickens in China reported in previous studies (34, 36). Since the replacements of key amino acid sites in VP1 between the vaccine sample JS2105A and reference strain AB1K were completely consistent. Therefore, the isolates JS2105A and JS2105B in this study may be mixed as contaminants in various vaccines to restore their virulence in the host (33). Of note, RDP4 software (RDP, GENECONV, Bootscan, MaxChi, Chimera, SiScan, and 3seq) and Simplot 3.51 software were used to assess the probability of genotype recombination of the 20 CIAV isolates and the 42 reference strains. However, the evaluation showed that there were potential recombination events in the reference strains (data not shown) instead of the 20 isolates, indicating that the variation of the isolates mainly due to natural mutations. Whether the genetic mutations in these isolates participate in the generation of new virus lineages needs to be further evaluated.

In summary, our study provided that the epidemiological data of CIAV in Jiangsu province of China during 2020–2022, which indicated a severe CIAV infection status in poultry industry. Notably, in clinical, it is necessary to pay more attention to the detection of CIAV in live vaccines to avoid CIAV infections caused by vaccination. Besides, the increasing isolation of CIAV strains with highly pathogenic characteristics is also worth our vigilance. Taken together, our study provides new insights into the epidemiology and effective strategies for the prevention and control of CIAV in China.

## Data availability statement

The data presented in the study are deposited in the GenBank repository, accession number PP354989-PP355008.

## Ethics statement

Ethical approval was not required for the studies on animals in accordance with the local legislation and institutional requirements because only commercially available established cell lines were used.

## Author contributions

JZ: Investigation, Writing – original draft. LM: Investigation, Writing – original draft. TL: Data curation, Investigation, Writing – original draft, Writing – review & editing. QX: Investigation, Writing – review & editing. ZW: Data curation, Methodology, Writing – review & editing. AQ: Data curation, Writing – review & editing. JY: Data curation, Methodology, Writing – original draft, Writing – review & editing. HS: Funding acquisition, Methodology, Writing – review & editing. SW: Writing – original draft, Writing – review & editing.
