# Supplementary material for: Isolation and genomic characterization of chicken infectious anemia virus in Jiangsu province of China during 2020–2022
Source: Front Vet Sci. 2024 Mar 14;11:1378120. doi: 10.3389/fvets.2024.1378120 (PMC10977075; doi:10.3389/fvets.2024.1378120)
Supplement: Supplementary file 1 [file Table_1.DOCX]

**Supplementary Table**

| Co-infection | Positive samples |
| --- | --- |
| CIAV+ALV-J | 4 |
| CIAV+ALV-K | 7 |
| CIAV+FAdV-4 | 5 |
| CIAV+ALV-J+ALV-K | 1 |
| CIAV+ALV-K+MDV | 2 |
| CIAV+REV+ARV+ALV-K+FAdV-8 | 1 |

sTable1 Co-infection detection of 36 CIAV-positive samples
